# Phosphate solubilizing microorganism: a green measure to effectively control and regulate heavy metal pollution in agricultural soils

**DOI:** 10.3389/fmicb.2023.1193670

**Published:** 2023-06-26

**Authors:** Xin Hu, Haoming Chen

**Affiliations:** School of Environmental and Biological Engineering, Nanjing University of Science and Technology, Nanjing, China

**Keywords:** heavy metals, bioremediation, phosphate solubilizing microorganisms, agricultural soils, food security

## 1. Introduction: heavy metals (HMs) pollution will seriously affect crop growth by destroying soil ecosystems

The acceleration of industrialization and urbanization will inevitably lead to HMs pollution entering the environment. Especially in the agricultural environment, farming, fertilization, irrigation, and other agricultural activities can result in a high concentration of HMs in the soil, causing most of the HMs become more active, thus inevitably being absorbed by crops (DalCorso et al., 2013). HMs have become one of the most serious pollutants to affect crops due to their high toxicity, concealment, and agglomeration. HMs can negatively affect crop growth, biomass, and photosynthesis by inhibiting enzyme function, destroying nucleic acid structure, and interfering with the uptake of phytonutrients, thus posing a threat to sustainable food production. Additionally, the high content of HMs in the soil is also a challenge to the safety of agricultural products. Excessive intake of foods containing HMs will cause irreversible harm to human health (Qin et al., 2021).

Rhizosphere is the key for plants to absorb nutrients and trace elements, and it is the interface of soil-plant-microorganism interactions. Heavy metal ions in the soil must enter the plant's body through the plant roots. As the nearest neighbor to plants, root microorganisms improve soil structure and soil fertility by participating in the formation and transformation of soil humus, the circulation of nutrients in the soil, etc. At the same time, root microorganisms can also secrete plant hormones to promote the absorption and utilization of nutrients by crops, and increase the root growth and biomass of crops (Etesami and Maheshwari, 2018; Manoj et al., 2020). However, high concentrations of HMs can cause abiotic stress by inducing microbial metabolic disorders (Wyszkowska et al., 2013), such as protein denaturation, cell membrane disintegration, change enzyme specificity, destroy cell function, and DNA structure (Abdu et al., 2017; Jacob et al., 2018), thereby disrupting the structure and function of the original microbial community. It is worth noting that changes in root microbial structure and quantity caused by HMs stress can seriously affect the ecological balance of the root system, leading to the decline of crop growth and the quality of agricultural products (Shen et al., 2019). Therefore, in order to ensure food security and human health, it is urgent to seek suitable measures (soil improvement and microbial community regulation) to remediate HMs pollution in farmland soil.

## 2. Microbial remediation technology is the best measure for HMs pollution suitable for agriculture

At present, the remediation technologies for HMs pollution in soil mainly include physical remediation, chemical remediation, and biological remediation. HMs in agricultural soil have a large contaminated area, but the peculiarities of crop cultivation dictate that traditional repair techniques with secondary pollution risks cannot be used. For example, long-term chemical fixation remediation (cement, asphalt, and other adhesives) will not only damage the integrity of the soil, but also have the risk of secondary pollution (Liu et al., 2018). In addition, the cost of traditional physical and chemical remediation technology is relatively high, and it is easy to cause secondary pollution, damage the soil ecological environment, make the cultivated land lose value, and reduce crop yield (Lin et al., 2022). In contrast, bioremediation is an efficient remediation technology, which can overcome many disadvantages of physical and chemical technology. Some studies have shown that bioremediation has good effect, low cost, small environmental interference, and no secondary pollution (Jacob et al., 2018). Among them, functional microorganisms can repair heavy metal pollution through bioaccumulation, biosorption, biotransformation, and other mechanisms (Mao et al., 2022), and can improve soil environment and promote crop growth through secretions, thus avoiding potential threats to human health and the environment caused by pollutant leakage (Khan et al., 2009). In particular, microbial remediation in the agri-environment must not only be effective in preventing and controlling pollution by HMs, but must also have the ability to improve crop yields.

## 3. The remediation function of HMs pollution by phosphate solubilizing microorganisms (PSM) is the key to ensuring food production and safety at the same time

### 3.1. PSM can effectively reduce the harm of HMs pollution in soil

**Figure 1:**
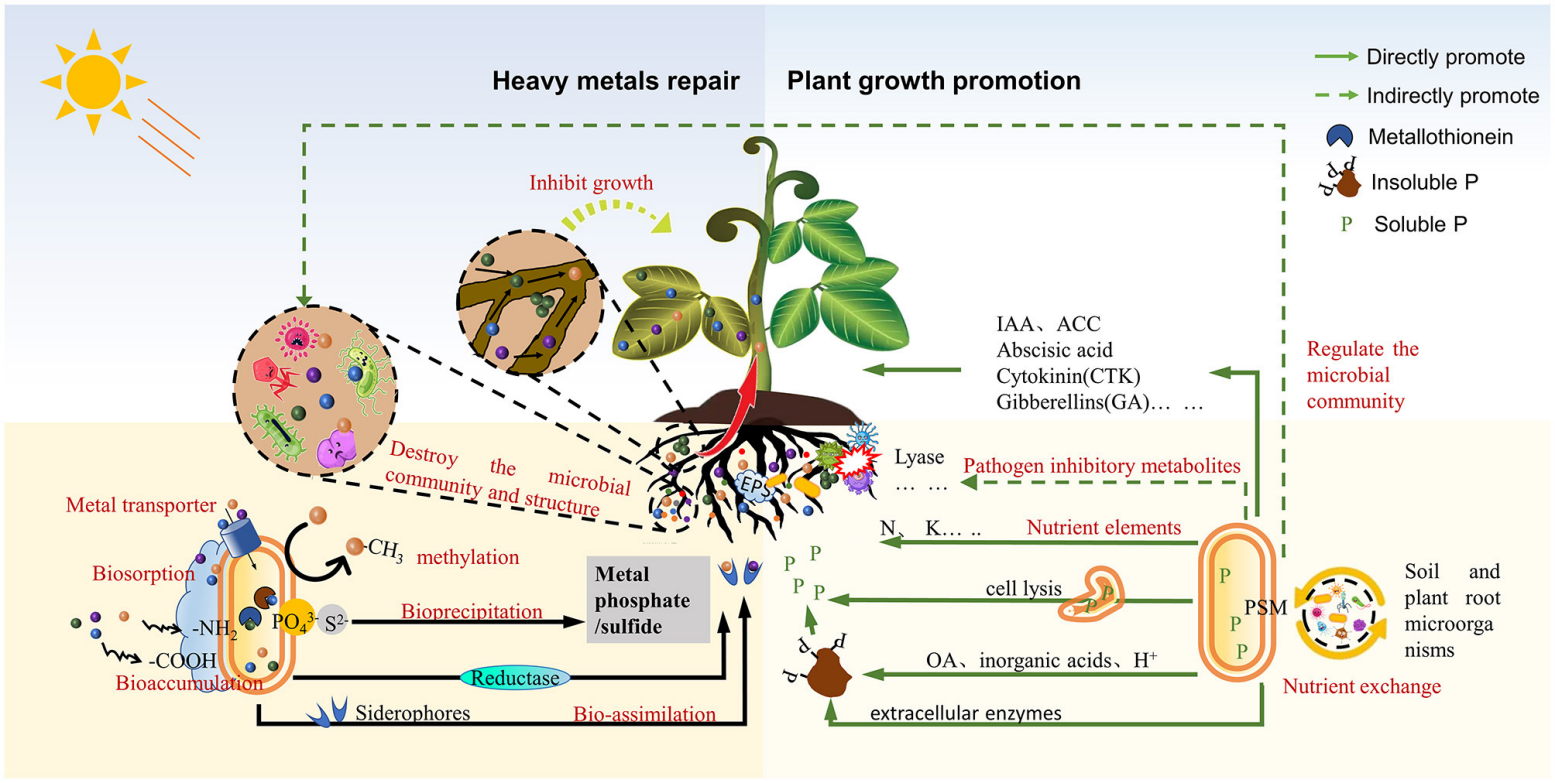
Schematic diagram of the mechanism of phosphate solubilizing microorganisms to repair soil heavy metals pollution and promote crop growth.

High concentrations of HMs are the most important limiting factor in the remediation of soil heavy metal contamination, so it is extremely important to screen for metal-resistant microorganisms. PSMs are functional microorganisms that promote the growth of plant rhizospheres and are known for their ability to release the P elements required by plants from insoluble phosphates. PSM plays a vital role in all three main processes that affect the soil phosphorus content [dissolution-precipitation, adsorption-desorption, and mineralization-immobilization (Sharma et al., 2013)]. It is worth noting that PSMs with high heavy metal resistance can alleviate HMs toxicity and the accumulation of HMs in plants through a range of mechanisms (Ahemad, 2015; Chen et al., 2023; Figure 1). In general, the tolerance of PSM to HMs is related to the isolation environment of the strain. For example, Jiang et al. solated *Burkeella* J62 with phosphorus-dissolving ability from heavy metal contaminated soil and found it has strong resistance to Cd (2,000 mg/L) and Pb (1,000 mg/L; Jiang et al., 2008). Teng et al. (2019) found that all 53 strains (e.g., *Leclercia adecarboxylata* and *Pseudomonas putida*) isolated from heavy metal contaminated soil were resistant to Pb. Therefore, heavy metal contaminated areas are an important source of heavy metal resistant phosphate solubilizing microbial strains, and heavy metal resistant PSM isolated from mining areas have great application potential in heavy metal contaminated soil remediation.

PSM mainly remediates heavy metal contaminated soil through bioadsorption and enrichment, biotransformation, and bioprecipitation (Chen et al., 2023). Firstly, PSM can reduce the bioavailability of HMs by synthesizing metal-binding proteins (such as metallothionein) and releasing siderophores to chelate HMs and accumulate them in the cells (Gupta and Kumar, 2017). Secondly, the anionic functional groups (such as sulfhydryl, carboxyl, hydroxyl, etc.) present on the surface of PSM can bind HMs through ion exchange, complexation, and other methods to achieve the purpose of adsorption (Priya et al., 2022). In addition, PSM can also utilize exopolysaccharides to complex and immobilize HMs and reduce toxicity (Naveed et al., 2019). In addition, PSM can metabolize and produce oxidoreductases to convert high toxic valence HMs in soil into low toxic valence states, and can also eliminate volatile metal derivatives produced by methylation from soil ecosystems through evaporation (Wang et al., 2022). Finally, the secretions of PSM (mainly organic acids, biological enzymes, etc.) are able to dissolve insoluble sources of phosphorus in the soil and produce specific anions (e.g., ${\text{PO}}_{4}^{3-}$, ${\text{SO}}_{4}^{2-}$). These PSM-generated anions are able to bind to heavy metals in the soil and convert them into heavy metal mineral precipitates (Naveed et al., 2019). This mechanism is also one of the key mechanisms of PSM for the remediation of HMs in soils. Different types of PSMs have different soil HMs remediation capacity and remediation mechanism, so more research is needed in the screening and identification of PSMs and the remediation application of agricultural HMs contaminated soils.

### 3.2. PSM improves the growth and health of crops in HMs contaminated soil

In addition to directly reducing the toxicity and bioavailability of HMs in soil to protect crops from HMs stress, PSM can also promote crop growth by improving the plant root soil ecosystem (Figure 1). Firstly, improving the physical and chemical properties of soil is the foundation for PSM to promote the growth and yield of crops. Due to the phosphate solubilization mechanism of PSM, inoculation with PSM can significantly increase the content of available trace elements such as P, K, N, and so on in soil (Liu et al., 2022), and improve soil beneficial characteristics such as SOM, enzyme activity, soil pH, and electrical conductivity (Liu et al., 2020), thereby affecting the growing environment of plants. Secondly, PSM inoculation can indirectly affect the diversity and richness of plant rhizosphere microbial communities, especially the nutrient exchange with beneficial microorganisms in crop roots, indirectly ensuring crop growth (Liu et al., 2020). Finally, the biological secretions of PSM can directly promote plant growth, which is the main reason why PSM is widely used in agriculture.

The benefits of PSM secretions on plant growth fall into three main categories: dissolved phosphorus sources, growth promotion, and disease inhibition. Among them, PSM increases the utilization of phosphorus in soil by plants through two secretions (Rawat et al., 2021). One is to dissolve insoluble inorganic phosphates by secreting protons and organic acids (acetic acid, citric acid, malic acid, etc.), inorganic acids. The second is to dissolve insoluble organic phosphates by secreting extracellular enzymes such as non-specific acid phosphatases, phytases, and carbon–phosphorus Lyases. Moreover, the bioavailable phosphorus fixed by PSM itself can also be used as a source of phosphorus for plants and other soil organisms after being released through cell lysis. The P released by PSM undoubtedly provides for the growth of agricultural plants. On the other hand, PSM can also directly secrete plant growth hormones (such as indole-3-acetic acid), abscisic acid, cytokinin, and gibberellin and other secretions to improve plant cell proliferation and root growth, stimulate plant lateral root proliferation, and regulate root ethylene levels, thereby promoting nutrient uptake by plants (Timofeeva et al., 2022). Additionally, PSM secretes 1-aminocyclopropane-1-carboxylate deaminase (ACC), which regulates the levels of ethylene in plant roots and mitigates the negative impact of excess ethylene on plant root growth and development (Ahemad, 2015). In addition to the aforementioned methods of directly promoting crop growth, PSM can also synthesize and release pathogen-inhibiting metabolites [mainly siderophores, plant hormones, and lyases (Vassilev et al., 2006; Saha et al., 2016)], which can inhibit the growth of plant pathogens, protect plants from plant pathogens, and indirectly promote crop growth. In conclusion, as a bioremediator of heavy metal contamination in soil, PSM can enhance crop health and nutritional status while remediating heavy metal contamination. Thus, it is undoubtedly one of the optimal measures for agricultural soil protection.

### 3.3. Prospects

PSM can be used not only as a passivation remediation agent for HMs in agri-environment, but also as a biofertilizer to promote plant growth, which will become a new method for the safety and utilization of agricultural production in the future. For researchers, there is a need to further investigate the resistance mechanism of PSM to different HMs contamination, so as to improve the adaptability and remediation effect of PSM. At the same time, it is necessary to strengthen the monitoring and evaluation of the PSM remediation process in order to better grasp the remediation application technology. During the research process, attention should also be paid to environmental protection and ecological balance to ensure that microbial remediation will not cause negative effects on the ecological environment. Examples include whether certain phosphate solubilizing microorganisms inoculated as exogenous microorganisms in the soil may disrupt the structure and function of the native microbial community, or whether they may inhibit beneficial microorganisms in the root system and cause disease in some plants, etc. Moreover, the combination of microbial remediation technology with other technologies, such as soil conditioners and biochar, to play a synergistic role will also be the future research direction.

## Author contributions

XH and HC conceived and wrote the paper. All authors contributed to the article and approved the submitted version.
